# Charge Transport and Thermoelectric Properties of Bornite with Fe-Site Off-Stoichiometry

**DOI:** 10.3390/ma19061252

**Published:** 2026-03-22

**Authors:** Hyemin Oh, Seungmin Lee, Hyeon-Sik O, Il-Ho Kim

**Affiliations:** Department of Materials Science and Engineering, College of Engineering, Korea National University of Transportation, Chungju 27469, Republic of Korea; ohm050510@naver.com (H.O.); sunglassmin1@naver.com (S.L.); ody644@naver.com (H.-S.O.)

**Keywords:** thermoelectric, bornite, non-stoichiometry, off-stoichiometry

## Abstract

The effects of Fe non-stoichiometry on crystal structure, microstructural evolution, and thermoelectric transport properties were systematically investigated in bornite (Cu_5_Fe_1+y_S_4_; −0.06 ≤ y ≤ 0.06) synthesized by mechanical alloying followed by hot pressing. X-ray diffraction analysis confirmed the formation of a single-phase orthorhombic bornite structure over the entire composition range. Anisotropic lattice distortion was observed with increasing Fe non-stoichiometry, manifested as contraction along the a-axis and expansion along the b- and c-axes, with a non-linear dependence on composition. Crystallite sizes estimated from Lorentzian peak fitting increased from 64.1 nm for the stoichiometric composition to 70.6–76.3 nm for Fe-deficient samples and 73.2–90.9 nm for Fe-excess samples. Hall-effect measurements revealed p-type semiconducting behavior for the stoichiometric composition, degenerate p-type transport with increased hole concentration under Fe-deficient conditions, and a transition to n-type behavior with reduced carrier mobility under Fe-excess conditions. While Fe-deficient samples retained high electrical conductivity and positive Seebeck coefficients, Fe-excess samples exhibited negative Seebeck coefficients at low temperatures with sign reversal at elevated temperatures. As a consequence, the power factor of Fe-deficient samples was enhanced by approximately 20–30% relative to the stoichiometric composition. In addition, the total thermal conductivity remained below 0.8 W·m^−1^·K^−1^ for all samples, and Fe non-stoichiometry effectively suppressed lattice thermal conductivity. Consequently, the Cu_5_Fe_0.94_S_4_ composition achieved a maximum dimensionless figure of merit of ZT = 0.61 at 673 K, representing a performance enhancement of approximately 30–70% compared with the stoichiometric composition (ZT = 0.36 at 673 K and 0.47 at 723 K).

## 1. Introduction

Thermoelectric conversion technology is a solid-state energy solution that directly converts waste heat into electricity and has demonstrated considerable potential for use in a wide range of industrial applications owing to its high reliability and excellent durability [1]. In recent years, the development of low-cost thermoelectric materials that deliver high performance while minimizing the use of toxic or scarce elements has become increasingly important to ensure both environmental and economic sustainability. In this context, Cu-based chalcogenides have attracted significant attention as promising candidates that satisfy these requirements. Their appeal arises from earth-abundant constituent elements, low toxicity, and a high degree of tunability in defect chemistry and composition, which has motivated extensive efforts to optimize their thermoelectric performance. In particular, numerous studies have focused on bornite (Cu_5_FeS_4_), aiming to enhance thermoelectric properties by controlling charge transport and thermal conductivity through defect engineering and compositional tuning [2,3,4,5].

Bornite, a natural sulfide mineral composed of copper, iron, and sulfur, is considered a promising thermoelectric material for large-scale production owing to its use of low-cost, non-toxic elements and relatively simple synthesis routes. This compound can be synthesized by various methods, including solid-state reactions [6,7], mechanochemical synthesis [3,4,8], and one-pot solution-based techniques [9,10], demonstrating excellent scalability for low-temperature and cost-effective processing. In addition, bornite has been reported to exhibit an intrinsically low lattice thermal conductivity of approximately 0.4–0.5 W·m^−1^·K^−1^ at room temperature [2,3,11] and to undergo a cation order–disorder phase transition in the temperature range of 473–543 K [12,13,14]. Such structural complexity and phase transition behavior not only effectively suppress lattice thermal conductivity by enhancing phonon scattering but also provide a means to regulate charge transport, thereby improving thermoelectric performance in the intermediate temperature range.

Recent studies have demonstrated that controlling non-stoichiometry plays a crucial role in enhancing the thermoelectric performance of bornite-based materials. In particular, modulation of Cu or Fe deficiency and excess has been reported to improve the power factor and ZT by regulating carrier concentration and mobility, while simultaneously reducing lattice thermal conductivity through enhanced phonon scattering [5,6,11,15]. Strategies for optimizing the thermoelectric performance of bornite can be broadly classified into three categories: (i) control of defects and microstructure through processing conditions; (ii) optimization of composition and doping based on band structure and scattering engineering; and (iii) suppression of lattice thermal conductivity via structural engineering. For example, ball milling-based mechanical alloying enables concurrent optimization of electrical conductivity and lattice thermal conductivity by introducing a high density of point defects, lattice strain, and grain refinement [3,4,8]. In addition, transition-metal doping has been widely adopted as an effective approach to balance the power factor and lattice thermal conductivity by precisely tuning carrier concentration, effective mass, and scattering mechanisms [4,16]. The formation of twin boundaries and nanostructures provides strong phonon-scattering centers while minimizing carrier mobility degradation, and excellent thermoelectric performance has been reported in materials with high-density twin structures and in large-scale bulk samples synthesized via colloidal routes [11,17,18,19]. Finally, Se substitution has been proposed as an effective strategy to achieve high thermoelectric performance with improved thermal stability in the intermediate temperature range by simultaneously optimizing the band structure, carrier concentration, and carrier mobility [7,14].

However, a quantitative understanding of phase transition stability, long-term durability, and synthesis-condition-dependent variations in crystal structure and charge transport properties—particularly from the perspective of defect chemistry—remains insufficient. To maximize the performance of bornite-based thermoelectric materials, it is essential to elucidate the atomic-scale distribution of Cu and Fe, the presence and evolution of vacancy concentrations, and their correlations with crystal structure and charge transport behavior. In this work, the thermoelectric performance of bornite-based materials was optimized by systematically establishing a range of Fe non-stoichiometry. Accordingly, Cu_5_Fe_1+y_S_4_ samples (−0.06 ≤ y ≤ 0.06) were synthesized using mechanical alloying followed by hot pressing. The effects of Fe deficiency and excess on phase evolution, lattice parameters, crystallite characteristics, charge transport, and thermoelectric performance were quantitatively compared and analyzed. Furthermore, by comparing the present results with previously reported data on stoichiometric and non-stoichiometric bornite, strategies for the co-optimization of carrier concentration, mobility, and phonon scattering were discussed, and the potential for further performance enhancement in bornite-based thermoelectric materials was evaluated.

## 2. Experimental Procedure

### 2.1. Sample Preparation

Non-stoichiometric bornite (Cu_5_Fe_1+y_S_4_; −0.06 ≤ y ≤ 0.06) samples were prepared from high-purity elemental powders. Copper (Cu, 99.9%, <45 µm), iron (Fe, 99.9%, <53 µm), and sulfur (S, 99.99%, <75 µm) powders were used as starting materials. The elements were accurately weighed according to the target compositions and pre-mixed using a rotary mixer to ensure compositional homogeneity. A total of 20 g of the mixed powder was loaded into a 500 mL hardened steel jar together with stainless steel balls of 5 mm diameter (total mass: 400 g), corresponding to a ball-to-powder ratio (BPR) of 20:1. Mechanical alloying (MA) was carried out under an argon atmosphere using a planetary ball mill (Pulverisette 5, Fritsch GmbH, Idar-Oberstein, Germany) operated at 350 rpm for 6 h. The mechanically alloyed powders were subsequently loaded into a graphite mold and consolidated by hot pressing (HP; JM-HP20, Jeongmin Industrial Co., Ltd., Seoul, Republic of Korea) at 723 K under a uniaxial pressure of 70 MPa for 2 h. The applied pressure was maintained throughout the sintering process, after which the samples were slowly cooled to room temperature under vacuum. The MA–HP processing conditions employed in this study were determined based on previously reported optimal preparation conditions for stoichiometric bornite [20,21].

### 2.2. Analysis of Crystal Structure and Microstructure

The crystalline phases were analyzed using an X-ray diffractometer (XRD; D8 Advance, Bruker AXS GmbH, Karlsruhe, Germany) with Cu Kα radiation. The measurements were conducted over a 2θ range of 10–90° with a step size of 0.02° and a counting time of 0.4 s per step. The obtained diffraction patterns were analyzed by Rietveld refinement using TOPAS software (v4.1, Bruker, Billerica, MA, USA). The relative density was calculated as the ratio of the measured density, determined from the sample mass and volume, to the theoretical density of bornite (4.9 g·cm^−3^) [22]. The crystallite size was estimated from the full width at half maximum (FWHM) of the diffraction peaks using Lorentzian peak fitting. The microstructures of polished cross-sections were examined using scanning electron microscopy (SEM; Prisma E, Thermo Fisher Scientific Inc., Waltham, MA, USA). The elemental composition of the stoichiometric Cu_5_FeS_4_ sample has been previously confirmed by energy-dispersive X-ray spectroscopy (EDS; Quantax 200, Bruker AXS GmbH, Karlsruhe, Germany) in our earlier work [20].

### 2.3. Measurement of Electrical and Thermal Properties

Charge transport properties were measured using a Hall measurement system (Model 7065, Keithley Instruments LLC, Solon, OH, USA) using the van der Pauw configuration at room temperature. Within the single parabolic band approximation, the carrier concentration (n) and mobility (μ) were calculated using the relations n = 1/(e·R_H_) and μ = σ·R_H_, respectively, where e is the elementary charge, R_H_ is the Hall coefficient, and σ is the electrical conductivity. The Seebeck coefficient (α) and electrical conductivity were measured simultaneously over the temperature range of 323–723 K using a thermoelectric property measurement system (ZEM-3, Advance Riko Inc., Yokohama, Japan) under a helium atmosphere. Thermal diffusivity (D) was measured using a laser flash apparatus (LFA717 HyperFlash, Netzsch GmbH, Selb, Germany) under vacuum conditions, and the specific heat capacity (c_p_) was estimated using the Dulong–Petit approximation. The thermal conductivity (κ) was then calculated according to κ = d·c_p_·D, where d is the sample density. Finally, the dimensionless figure of merit (ZT = α^2^·σ·κ^−1^·T) was evaluated in the temperature (T) range of 323–723 K using the power factor (PF = α^2^·σ), together with the thermal conductivity (κ).

## 3. Results and Discussion

**Figure 1:**
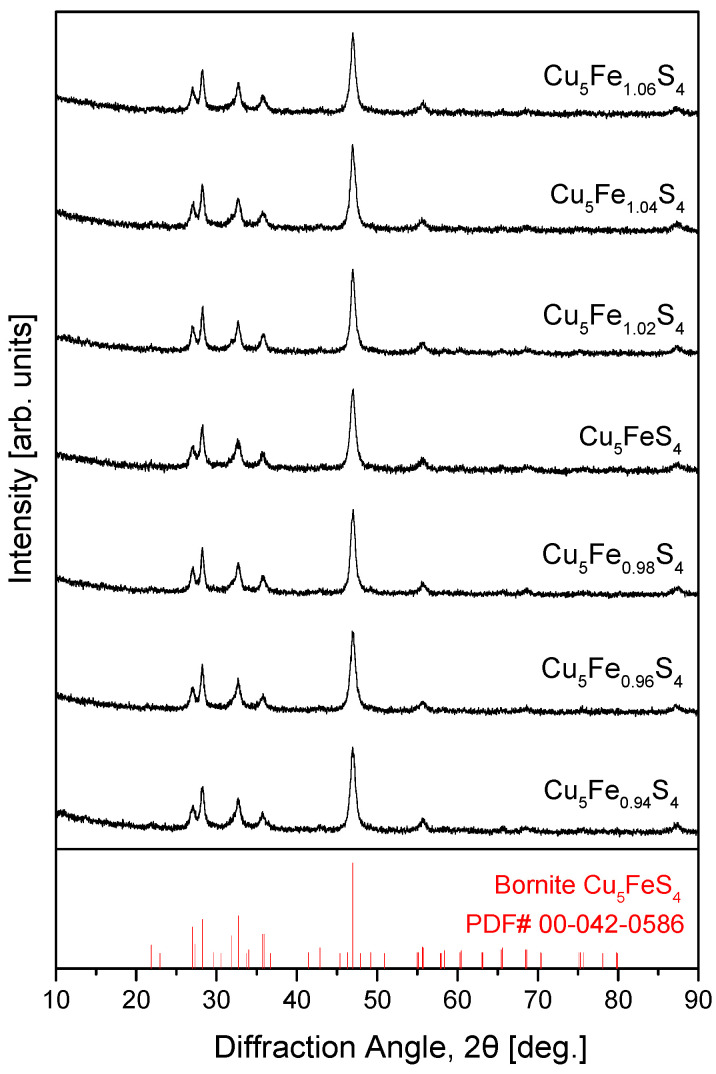
XRD patterns of mechanically alloyed Cu_5_Fe_1+y_S_4_ (−0.06 ≤ y ≤ 0.06) powders. All reflections are consistent with the orthorhombic bornite, and no secondary phases are detected. For comparison, the data for the stoichiometric Cu_5_FeS_4_ sample were taken from our previous study [21].

**Figure 2:**
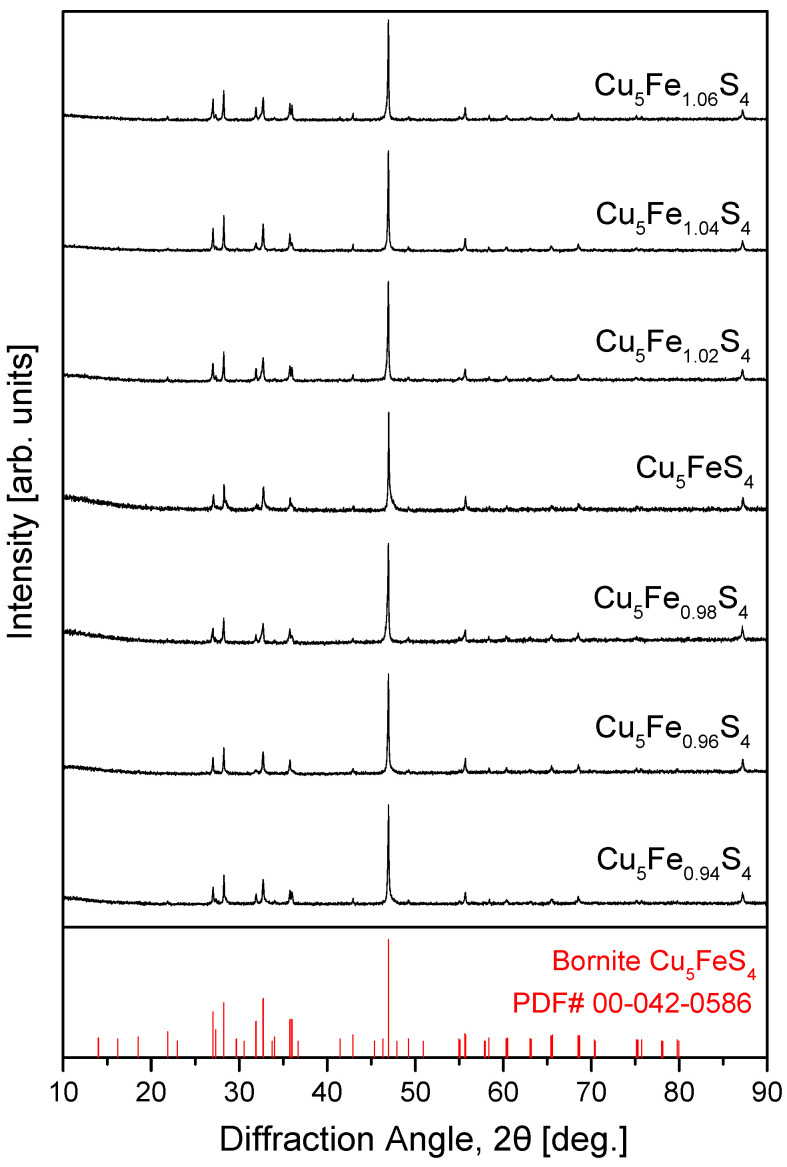
XRD patterns of hot-pressed Cu_5_Fe_1+y_S_4_ (−0.06 ≤ y ≤ 0.06) samples. Single-phase orthorhombic bornite is retained for all compositions in agreement with the standard pattern. For comparison, the data for the stoichiometric Cu_5_FeS_4_ sample were taken from our previous study [21].

Figure 1 presents the XRD patterns of Cu_5_Fe_1+y_S_4_ (−0.06 ≤ y ≤ 0.06) powders prepared via the MA process. The diffraction peaks of all samples corresponded well to the standard pattern of bornite (PDF# 00-042-0586), indicating that a single phase was stably formed directly during the MA process. No secondary phases, such as Cu_2−x_S or Fe–S–based sulfides, were detected within the detection limit of the instrument. This result is consistent with previous reports indicating that the MA process effectively suppresses residual Cu_2−x_S, which is often observed in conventional solid-state reactions, and significantly shortens the synthesis time [3,4,8]. Accordingly, the reproducible formation of a single phase was confirmed over the entire composition range investigated in this study. The stoichiometric composition (y = 0) exhibited diffraction peaks characteristic of low-temperature orthorhombic bornite (LO-Bornite, space group Pbca), and both Fe-deficient (y < 0) and Fe-excess (y > 0) compositions exhibited identical diffraction patterns. This indicates that the non-stoichiometric compositions formed the bornite lattice without inducing phase separation or the formation of additional crystalline phases. Furthermore, the generally broadened diffraction peaks observed in the MA-synthesized powders are attributed to the formation of nanocrystallites and lattice strain, which are typical features of MA processes. The compositional dependence and detailed variations in lattice parameters are discussed in the subsequent analysis of the sintered samples (Figure 2 and Table 1).

Figure 2 presents the XRD patterns of the hot-pressed Cu_5_Fe_1+y_S_4_ (−0.06 ≤ y ≤ 0.06) samples. All samples are in good agreement with the standard pattern of LO-Bornite (PDF# 00-042-0586), and no secondary phases were detected. Furthermore, neither intermediate-temperature cubic bornite (IC-Bornite, space group $Fm\bar{3}m$) nor high-temperature cubic bornite (HC-Bornite, space group $Fm\bar{3}m$) was formed, even after the high-temperature HP process. Compared with the MA powders, the diffraction peaks of the sintered samples became noticeably sharper, accompanied by reduced peak broadening. This behavior is attributed to the relaxation of residual lattice strain introduced during the MA process, along with subsequent grain growth during HP. Such strain relaxation and crystallite growth are consistent with trends generally reported for high-energy milling followed by HP processing [3,4]. Notably, the single-phase LO-Bornite structure, which is thermodynamically stable at room temperature, was preserved across all compositions despite the introduction of Fe non-stoichiometry, indicating that variations in Fe content (−0.06 ≤ y ≤ 0.06) did not induce phase separation or structural transitions. The diffraction patterns were further analyzed by Rietveld refinement with orthorhombic bornite (space group Pbca) as the structural model. The scale factor, background, zero shift, lattice parameters, and peak profile parameters were refined. For the six refined patterns, the refinement converged with reliability factors of R_exp_ = 5.51–6.58%, R_p_ = 5.70–6.85%, R_wp_ = 7.31–8.72%, and χ^2^ = 1.23–1.51. These consistently low residuals and near-unity χ^2^ values confirm good refinement quality over the entire composition range and support the reliability of the refined lattice parameters listed in Table 1.

As shown in Table 1, the HP-sintered bodies achieved very high relative densities of 98.4–100.0% across all compositions, indicating the formation of nearly fully dense samples. The lattice parameters (a, b, and c), obtained from lattice refinement, exhibited systematic compositional trends, indicating anisotropic lattice distortion associated with Fe non-stoichiometry. In Fe-deficient compositions, the increases in carrier concentration and electrical conductivity observed in Hall measurements may be related to changes in the effective defect population within the bornite lattice, possibly involving Cu-site vacancies. Such defect-related lattice disorder may contribute to anisotropic lattice relaxation, which is consistent with the experimentally observed contraction along the a-axis together with slight expansion along the b- and c-axes. Conversely, in Fe-excess compositions, the p–n transition and reduced carrier concentration may reflect different defect configurations associated with Fe non-stoichiometry, which could be accompanied by partial relaxation of lattice strain. These trends are consistent with the proposed occupation model of bornite, in which Fe occupies tetrahedral sites and Cu partially occupies split sites, as well as with previous reports suggesting that variations in Cu^2+^ content and vacancy population may influence lattice parameters and superlattice reflections [6,22].

In bornite, Cu^2+^ ions have been suggested in previous studies to contribute to charge compensation and structural stability. Upon the introduction of Fe non-stoichiometry, the defect chemistry of bornite may be influenced, possibly involving changes in the Cu^+^/Cu^2+^ balance and the effective Cu-site vacancy population, as suggested in previous studies. Under Fe-deficient conditions, the observed transport behavior may be interpreted in terms of a possible increase in the effective vacancy population within the bornite lattice as part of the charge-compensation mechanism. Such defect-related lattice disorder may induce anisotropic lattice relaxation, which is consistent with the experimentally observed contraction along the a-axis together with slight expansion along the b- and c-axes. Previous studies have also suggested that Cu^2+^–S bonding can influence the electronic structure and transport characteristics of bornite, which is consistent with the observed p-type conduction. This interpretation is qualitatively consistent with previous reports indicating that the LO ↔ IC transition in bornite can occur during the thermal history of high-temperature sintering processes such as HP [3,4,15]. The crystallite size, extracted by Lorentzian fitting, was smallest for the stoichiometric composition (64.1 nm) and increased to 70.6–76.3 nm for Fe-deficient compositions and 73.2–90.9 nm for Fe-excess compositions.

**Figure 3:**
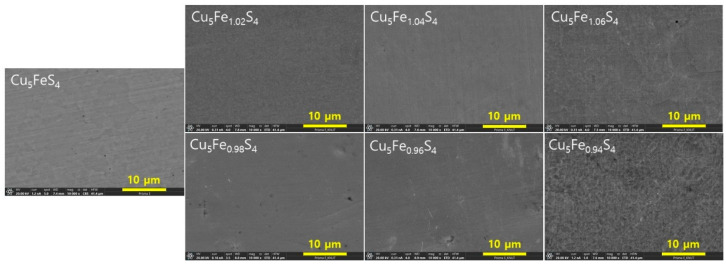
Cross-sectional SEM microstructures of Cu_5_Fe_1+y_S_4_ samples prepared by the MA–HP process. For comparison, the data for the stoichiometric Cu_5_FeS_4_ sample were taken from our previous study [21].

Figure 3 presents SEM microstructures of Cu_5_Fe_1+y_S_4_ samples sintered by HP from MA powders for compositional comparison. The stoichiometric composition (Cu_5_FeS_4_) exhibited a homogeneous and dense microstructure, whereas the Fe-deficient compositions showed subtle contrast variations and slight microstructural inhomogeneity. In contrast, the Fe-excess compositions were characterized by relatively coarse microstructural features and pronounced grain boundary contrast. These differences in microstructural appearance indicate that Fe non-stoichiometry influences the microstructural evolution during the HP sintering process. Although these microstructural features are qualitatively consistent with the crystallite size trends derived from XRD analysis, a direct quantitative comparison is not appropriate because the characteristic length scales probed by the two techniques are fundamentally different. Therefore, the SEM observations are discussed here mainly in terms of qualitative microstructural differences among the compositions rather than detailed defect-related mechanisms. In metallic systems and relatively simple sulfides, an increase in vacancy concentration has often been associated with enhanced microstructural coarsening by providing pathways for atomic diffusion. However, in structurally complex chalcogenides such as bornite, excessive Cu vacancies have been reported to induce lattice distortion and defect clustering, thereby increasing local structural disorder [11,15].

**Figure 4:**
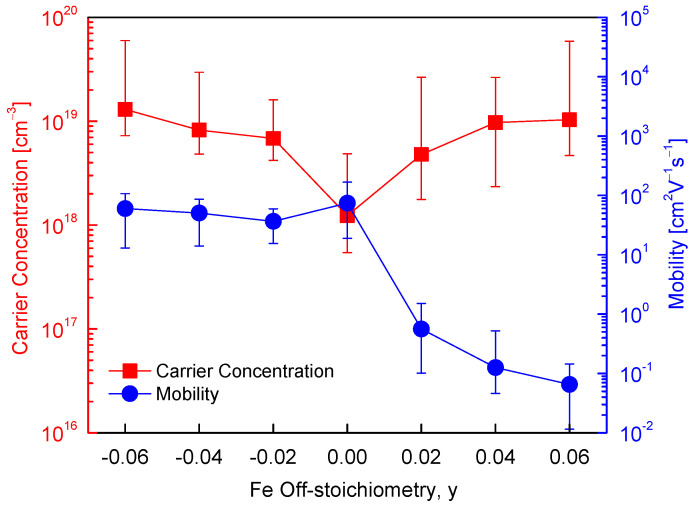
Composition dependence of carrier concentration and mobility obtained from Hall measurements. Fe-deficient compositions convert to heavily doped p-type with gradually reduced mobility, while Fe-rich compositions show n-type conversion with sharply decreased mobility. For comparison, the data for the stoichiometric Cu_5_FeS_4_ sample were taken from our previous study [21].

Figure 4 presents the Hall measurement results for Cu_5_Fe_1+y_S_4_. The stoichiometric composition (y = 0) exhibited p-type conduction behavior, as determined from the sign of the Hall coefficient obtained during the Hall measurement. The corresponding hole concentration and carrier mobility were 1.2 × 10^18^ cm^−3^ and 74 cm^2^·V^−1^·s^−1^, respectively. This relatively high carrier mobility is comparable to, or slightly higher than values reported for polycrystalline bornite-based materials and likely reflects the intrinsically favorable electronic transport characteristics of bornite under near-stoichiometric conditions with limited defect scattering. This behavior is consistent with the intrinsic p-type transport characteristics of bornite, which have often been interpreted in terms of the balance between Cu-site vacancies and Cu^2+^–Fe site occupation [6,22]. In Fe-deficient compositions (y < 0), the hole concentration increased to the order of 10^19^ cm^−3^, while the carrier mobility decreased to the range of 37–60 cm^2^·V^−1^·s^−1^. This behavior reflects a trade-off in which the increased hole concentration may be associated with changes in the effective defect population, possibly involving Cu-site vacancies under Fe-deficient conditions, whereas the mobility is reduced due to enhanced scattering from lattice disorder and defect-related scattering centers [6,8]. Conversely, in Fe-excess compositions (y > 0), the dominant carrier type transitioned to electrons, indicating n-type conduction. Although the electron concentration remained on the order of 10^19^ cm^−3^, the carrier mobility decreased markedly to a very low range of 10^−1^–10^−2^ cm^2^·V^−1^·s^−1^. This behavior may be associated with the formation of electron-donor antisite defects, ${Fe}_{Cu}^{˙˙}$, associated with excess Fe, accompanied by a simultaneous reduction in the Cu vacancy concentration ($V_{Cu}^{\prime }$). In addition, carrier mobility is further suppressed by the combined effects of alloy scattering, point defect scattering, and grain boundary scattering.

**Figure 5:**
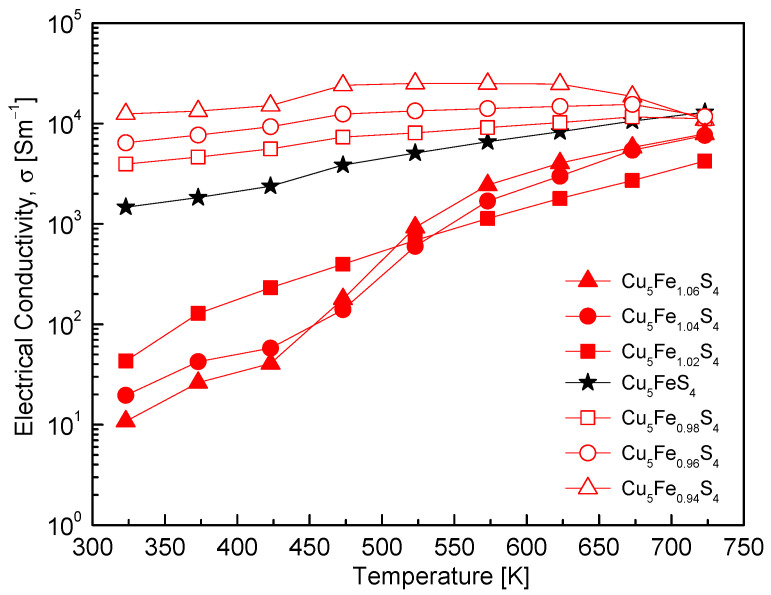
Temperature dependence of the electrical conductivity. Fe-deficient compositions exhibit higher values with weak temperature dependence, while Fe-rich compositions show low conductivity at low temperatures followed by an increase at high temperatures. For comparison, the data for the stoichiometric Cu_5_FeS_4_ sample were taken from our previous study [21].

Figure 5 shows the temperature dependence of the electrical conductivity (σ) for Cu_5_Fe_1+y_S_4_. The conduction behavior varies significantly with Fe composition, indicating a strong dependence on the charge transport characteristics of bornite. The stoichiometric composition exhibited semiconducting behavior, starting from approximately 1.5 × 10^3^ S·m^−1^ at 323 K and gradually increasing with temperature to reach 1.3 × 10^4^ S·m^−1^ at 723 K. This trend is consistent with the Hall measurement results shown in Figure 4, which indicate hole-dominant transport with moderate carrier mobility. The Fe-deficient compositions exhibited the highest σ over the entire temperature range, maintaining values on the order of 10^4^ S·m^−1^ even at 323 K. This behavior is consistent with p-type degenerate transport associated with an increased hole concentration. Such behavior is qualitatively consistent with the characteristics of bornite, in which the reduction in σ remains relatively limited despite enhanced defect-related and lattice-disorder scattering [8]. In contrast, the Fe-excess compositions exhibited very low σ values in the range of 10–10^2^ S·m^−1^ at 323–473 K but showed thermally activated behavior, with σ increasing rapidly with temperature to reach an intermediate level of approximately 10^3^ S·m^−1^ at 723 K. This behavior is related to reduced carrier mobility, possibly associated with increased defect-related scattering and microstructural disorder, together with the n-type transition (Figure 4). The convergence of σ to similar values at elevated temperatures for all compositions is attributed to the combined effects of the LO-Bornite ↔ IC-Bornite order–disorder transition, thermal activation of intrinsic carriers, and band structure readjustment, including changes in the effective mass. In summary, the Fe-deficient compositions exhibit hole-dominant conduction with high σ and weak temperature dependence, whereas the Fe-excess compositions display low σ, strong thermally activated behavior, and a clear p–n transition.

**Figure 6:**
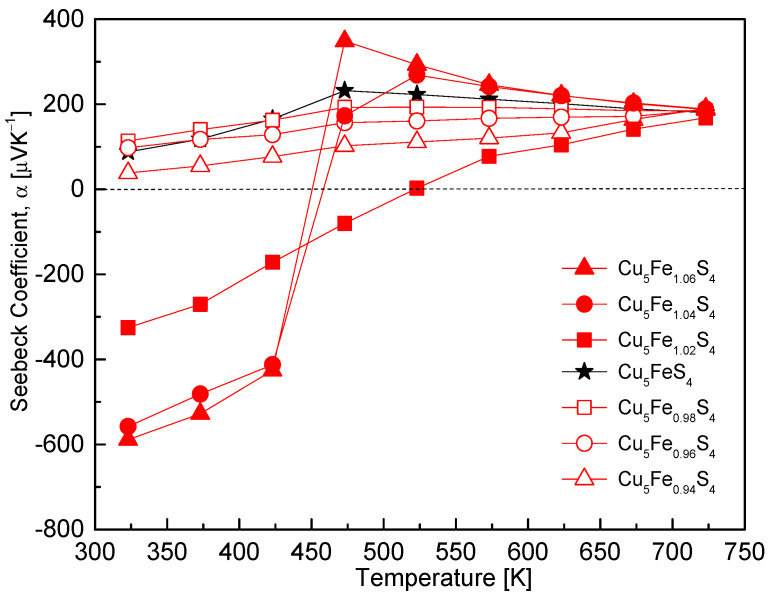
Temperature dependence of the Seebeck coefficient. Fe-deficient compositions maintain positive values across the full range, whereas Fe-rich compositions exhibit negative values at low temperatures, sign reversal near 450–550 K, and convergence to positive values at high temperatures. For comparison, the data for the stoichiometric Cu_5_FeS_4_ sample were taken from our previous study [21].

Figure 6 shows the temperature dependence of the Seebeck coefficient (α) for Cu_5_Fe_1+y_S_4_. Both the magnitude and temperature dependence of α vary significantly with Fe composition, reflecting systematic changes in charge transport behavior. The stoichiometric composition maintained positive α values ranging from 88 to 232 μV·K^−1^ over the temperature range of 323–723 K, indicating p-type dominant transport. This is consistent with the hole-dominant structure–transport relationship in bornite [6,22], and also consistent with previous reports showing that α–T characteristics can be systematically tuned through modifications of the band structure and defect chemistry induced by compositional changes [7,14]. The Fe-deficient compositions retained positive α values across the entire temperature range but exhibited slightly lower α values than the stoichiometric composition (38–113 μV·K^−1^ at low temperatures and 184–190 μV·K^−1^ at high temperatures), with α increasing gradually with temperature. This behavior is attributed to the increased hole concentration, which leads to a reduction in α in accordance with the Pisarenko relation (α ∝ m*·T·n^−1^), where m* is the effective mass [1]. Because the carrier mobility (Figure 4) varies only moderately with Fe deficiency, the contribution of effective mass variations to α is expected to be limited [8]. In contrast, Fe-excess compositions exhibited a pronounced decrease in electron mobility, suggesting an increase in the effective mass associated with a flattening of the electronic band dispersion, which leads to an increase in the absolute magnitude of α (negative values). These compositions showed large negative α values ranging from −588 to −325 μV·K^−1^ at low temperatures; however, the sign inverted in the temperature range of 450–550 K, converging to positive values of +168 to +189 μV·K^−1^ at high temperatures. The convergence of α to similar values at elevated temperatures for all compositions is attributed to the combined effects of band structure reconstruction and thermal activation of intrinsic carriers near the LO-Bornite ↔ IC-Bornite order–disorder phase transition [6].

**Figure 7:**
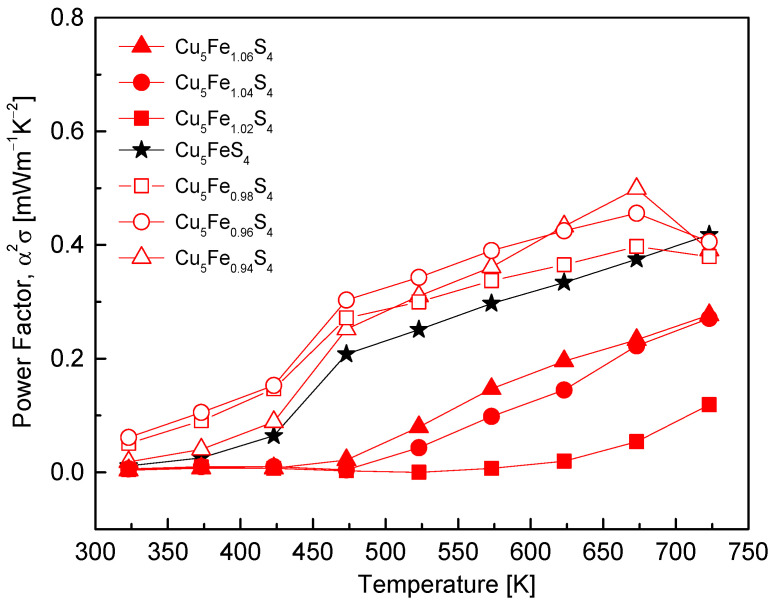
Temperature dependence of the power factor. The Fe-deficient compositions exhibit higher values than the stoichiometric composition, whereas the Fe-rich compositions remain low over the entire range. For comparison, the data for the stoichiometric Cu_5_FeS_4_ sample were taken from our previous study [21].

Figure 7 shows the temperature dependence of the power factor (PF) for Cu_5_Fe_1+y_S_4_ as a function of composition. The stoichiometric composition exhibited a gradual increase in PF over the entire measured temperature range, reaching 0.42 mW·m^−1^·K^−2^ at 723 K. This behavior is attributed to the combination of hole-dominant transport and moderate carrier mobility. The Fe-deficient compositions exhibited the highest PF values in the temperature range of 323–673 K, achieving a maximum PF of 0.50 mW·m^−1^·K^−2^ at 673 K. This enhancement arises from increased electrical conductivity due to higher carrier concentration while maintaining a reasonably large Seebeck coefficient [6,22]. In contrast, the Fe-excess compositions exhibited very low PF values at low temperatures due to their low σ. Even after the sign inversion of α in the temperature range of 450–550 K, the PF remained limited to 0.12–0.28 mW·m^−1^·K^−2^ (average ≈ 0.2 mW·m^−1^·K^−2^) at 723 K. This behavior is primarily attributed to the n-type transition induced by Fe excess and the degradation of electrical conductivity caused by severely reduced carrier mobility, leading to strong suppression of the overall α^2^·σ term. In summary, the Fe-deficient compositions achieved the most pronounced enhancement in PF by simultaneously achieving high σ and reasonable α, whereas the Fe-excess compositions exhibited markedly reduced σ and PF due to the n-type transition and enhanced defect-related scattering.

**Figure 8:**
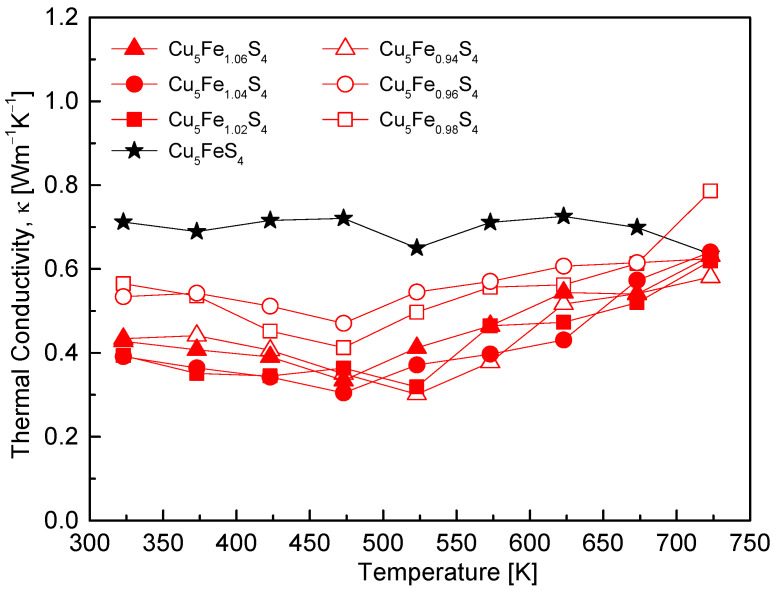
Temperature dependence of the thermal conductivity. All samples show ultralow thermal conductivity values, with Fe-deficient compositions exhibiting further suppression compared with the stoichiometric sample, while Fe-rich samples display reduced thermal conductivity values at low temperatures but an upturn at higher temperatures. For comparison, the data for the stoichiometric Cu_5_FeS_4_ sample were taken from our previous study [21].

Figure 8 shows the temperature dependence of the thermal conductivity (κ) for Cu_5_Fe_1+y_S_4_. For all compositions, κ values remained below 0.8 W·m^−1^·K^−1^, demonstrating the intrinsically low thermal conductivity characteristics of bornite. The stoichiometric composition exhibited relatively weak temperature dependence over the temperature range of 323–723 K, with κ varying from 0.64 to 0.73 W·m^−1^·K^−1^ and a broad minimum observed around 523 K, followed by a gradual increase at higher temperatures. This behavior is consistent with previous reports indicating a readjustment of phonon scattering mechanisms near the T_1_ phase transition [12,13]. The Fe-deficient compositions generally exhibited lower κ values than the stoichiometric composition; notably, the y = −0.06 sample reached a minimum κ of 0.30 W·m^−1^·K^−1^ at 523 K. This reduction is attributed to suppression of the lattice thermal conductivity (κ_L_) through enhanced phonon scattering by point defects and grain boundaries. However, κ increased again at elevated temperatures due to the rise in electronic thermal conductivity (κ_E_ ∝ σ·T) resulting from the high electrical conductivity. The Fe-excess compositions exhibited lower κ values overall, which may reflect enhanced phonon scattering arising from compositional fluctuations and lattice strain associated with Fe non-stoichiometry. Nevertheless, as κ_E_ increased with temperature owing to n-type conduction and higher σ, the total κ increased in the high-temperature region.

**Figure 9:**
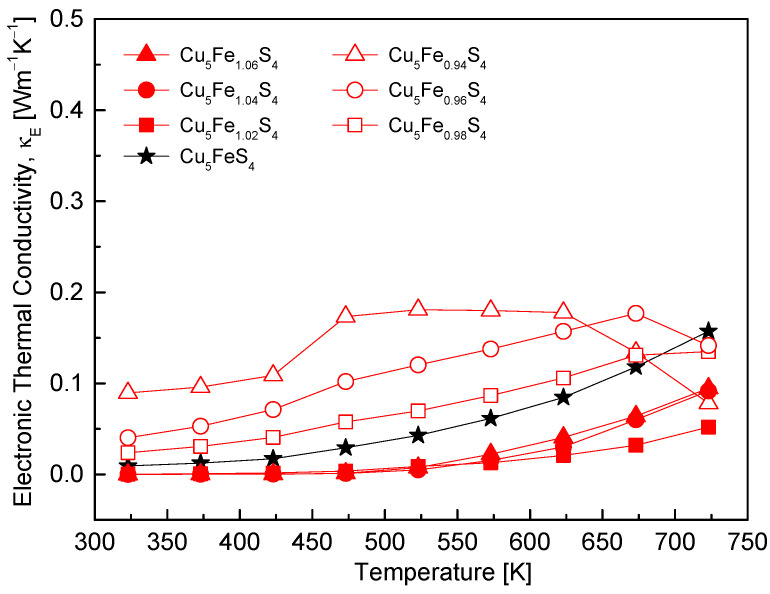
Temperature dependence of the electronic thermal conductivity. Fe-deficient samples maintain relatively high values due to enhanced electrical conductivity, whereas Fe-rich samples show suppressed values at low temperatures but approach similar levels at elevated temperatures. For comparison, the data for the stoichiometric Cu_5_FeS_4_ sample were taken from our previous study [21].

Figure 9 shows the temperature dependence of the electronic thermal conductivity (κ_E_) for Cu_5_Fe_1+y_S_4_. The electronic thermal conductivity was calculated using the Wiedemann–Franz law (κ_E_ = L·σ·T). The Lorenz number (L) was determined at each temperature using an empirical expression dependent on the Seebeck coefficient within the single parabolic band approximation, given by L [10^−8^ V^2^·K^−2^] = 1.5 + exp(−|α|/116) [23]. Accordingly, κ_E_ for each composition was calculated by incorporating the temperature-dependent L(T) values. Reflecting the intermediate electrical conductivity (Figure 5) and positive Seebeck coefficient (Figure 6), the stoichiometric composition exhibited typical p-type characteristics, with κ_E_ gradually increasing with temperature over the range of 323–723 K. The Fe-deficient compositions showed relatively high κ_E_ values across the entire temperature range owing to the increased hole concentration and high σ. In contrast, the Fe-excess compositions exhibited low κ_E_ values at low temperatures because σ was strongly suppressed by the n-type transition and a drastic reduction in carrier mobility; however, a gradual increase in κ_E_ was observed with increasing temperature as σ increased.

**Figure 10:**
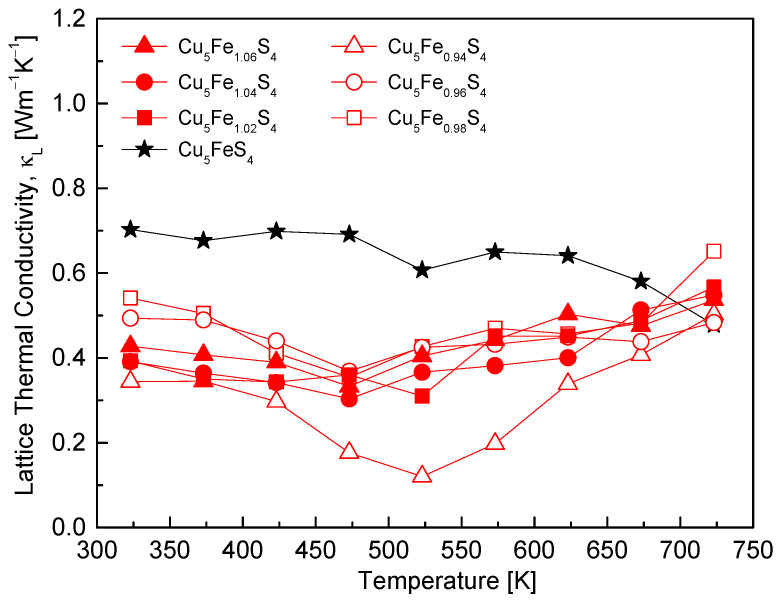
Temperature dependence of the lattice thermal conductivity. Fe off-stoichiometry enhances phonon scattering, leading to lower values than the stoichiometric composition, with Fe deficiency giving the strongest reduction. For comparison, the data for the stoichiometric Cu_5_FeS_4_ sample were taken from our previous study [21].

Figure 10 shows the temperature dependence of the lattice thermal conductivity (κ_L_) for Cu_5_Fe_1+y_S_4_ samples. Both the magnitude and temperature dependence of κ_L_ vary with Fe non-stoichiometry, demonstrating the compositional dependence of phonon scattering behavior. The stoichiometric composition exhibited moderate temperature dependence over the temperature range of 323–723 K, with κ_L_ values ranging from 0.48 to 0.70 W·m^−1^·K^−1^, reaffirming the intrinsically low lattice thermal conductivity of bornite. The Fe-deficient compositions generally exhibited lower κ_L_ values; notably, the y = −0.06 sample reached a minimum κ_L_ of 0.12 W·m^−1^·K^−1^ at 523 K, indicating the strongest phonon scattering. This reduction may be associated with the combined effects of grain boundary scattering and phonon scattering related to lattice disorder and compositional fluctuations. These scattering mechanisms originate from the refined microstructure and lattice strain introduced by the MA–HP process. Notably, the lowest κ_L_ value obtained in this study is remarkably low, even when compared with previously reported cases of thermal conductivity reduction in bornite systems. For example, Lashkari et al. [5] reported a reduced thermal conductivity of approximately 0.19 W·m^−1^·K^−1^ at 473 K relative to undoped samples by enhancing phonon scattering through mass mismatch and point defects induced by Ni doping. This result suggests that Fe non-stoichiometry alone can achieve ultralow lattice thermal conductivity comparable to that obtained via doping or nanostructuring. The Fe-excess compositions also exhibited lower κ_L_ values, typically in the range of 0.4–0.5 W·m^−1^·K^−1^, compared to the stoichiometric composition. For all compositions, a broad minimum in κ_L_ around 500 K followed by an increase at higher temperatures was observed, which can be qualitatively associated with changes in phonon scattering contributions near the T_1_ phase transition.

**Figure 11:**
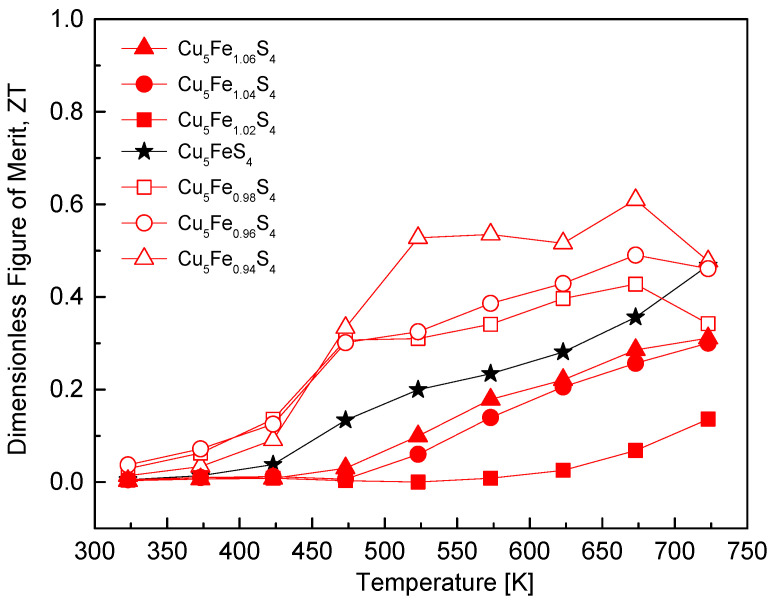
Temperature dependence of the dimensionless figure of merit (ZT). Fe-deficient compositions exhibit enhanced ZT across the measured range, whereas Fe-rich compositions show relatively lower values due to n-type conversion and reduced mobility For comparison, the data for the stoichiometric Cu_5_FeS_4_ sample were taken from our previous study [21].

Figure 11 shows the temperature dependence of the dimensionless figure of merit (ZT) for Cu_5_Fe_1+y_S_4_. The stoichiometric composition exhibited a ZT value of 0.005 at 323 K, which increased continuously with temperature to reach 0.47 at 723 K. The Fe-deficient compositions displayed higher ZT values over the entire temperature range; in particular, the y = −0.06 sample achieved a maximum ZT of 0.61 at 673 K. This enhancement is attributed to the combined effects of an improved power factor driven by increased hole concentration, effective suppression of lattice thermal conductivity induced by Fe non-stoichiometry and the MA–HP process. In contrast, the Fe-excess compositions exhibited markedly reduced ZT values at low temperatures in association with the transition to n-type conduction and the pronounced reduction in carrier mobility. Although ZT increased in the temperature range of 450–550 K due to the increase in the magnitude of the Seebeck coefficient together with the associated increase in electrical conductivity, the ZT values reached only 0.14–0.31 at 723 K, remaining inferior to those of the Fe-deficient compositions. This trend is consistent with previous studies. Zhang et al. [19] reported that Cu-rich/Fe-deficient bornite prepared via colloidal synthesis followed by spark plasma sintering (SPS) exhibited an enhanced ZT of approximately 0.56 at 690 K compared to the stoichiometric composition, which was attributed to increased carrier concentration arising from Cu excess and strong phonon scattering induced by nanocrystallites. Similarly, Zhang et al. [11] achieved a ZT of approximately 0.62 at 710 K by introducing high-density twin boundaries, where strong phonon scattering was coupled with preservation of the power factor through mobility retention. Furthermore, Zheng et al. [15] demonstrated a ZT of approximately 0.90 at 768 K in non-stoichiometric bornite (Cu_6.2_Fe_0.6_S_4_) synthesized via colloidal methods and SPS, which was attributed to increased hole concentration, retained mobility associated with the Cu-rich/Fe-deficient structure, and substantial suppression of lattice thermal conductivity by core–shell structures and high-density twin boundaries. These results suggest that the synergistic combination of simultaneous Cu/Fe non-stoichiometry control and nanostructuring enables higher thermoelectric performance than that achieved in the present study, which employed only Fe non-stoichiometry. In this work, although the power factor was enhanced through increased hole concentration, the overall improvement in ZT was constrained by concurrent mobility degradation as well as by increased electronic thermal conductivity at elevated temperatures. Consequently, further enhancement of the thermoelectric performance of bornite is expected to require quantitative control of Cu non-stoichiometry or simultaneous Cu/Fe non-stoichiometry.

## 4. Conclusions

The effects of Fe non-stoichiometry on the crystal structure, microstructure, and thermoelectric transport properties of bornite (Cu_5_Fe_1+y_S_4_; −0.06 ≤ y ≤ 0.06) synthesized by mechanical alloying followed by hot pressing were systematically investigated. A single-phase bornite structure was maintained across all compositions. The lattice parameters exhibited anisotropic variations characterized by contraction along the a-axis and expansion along the b- and c-axes for both Fe-deficient and Fe-excess compositions, with more pronounced lattice distortion observed under Fe-excess conditions. The crystallite size increased from 64.1 nm for the stoichiometric composition to an average of 72.5 nm for Fe-deficient compositions and 83.9 nm for Fe-excess compositions. In terms of charge transport, the stoichiometric composition exhibited p-type conduction, whereas Fe-deficient compositions showed degenerate p-type behavior driven by increased hole concentration, accompanied by a moderate reduction in carrier mobility. In contrast, Fe-excess compositions underwent a transition to electron-dominant conduction with a severe reduction in mobility. As a result, Fe-deficient compositions exhibited an enhanced power factor primarily due to their high electrical conductivity while maintaining a reasonable Seebeck coefficient. In addition, lattice thermal conductivity was effectively suppressed, leading to a significant improvement in the dimensionless figure of merit. Conversely, Fe-excess compositions exhibited inferior thermoelectric performance owing to the limited power factor associated with mobility degradation. These results indicate that Fe non-stoichiometry can influence carrier transport and phonon scattering behavior in bornite-based thermoelectric materials. Further improvements in thermoelectric performance may require more systematic control of cation non-stoichiometry and microstructural features.

## Figures and Tables

**Table 1:** Relative density, lattice constants, and crystallite size of hot-pressed Cu_5_Fe_1+y_S_4_ samples. For comparison, the data for the stoichiometric Cu_5_FeS_4_ sample were taken from our previous study [21].

Specimen	Relative Density [%]	Lattice Constant [nm]			Crystallite Size [nm]
		a	b	c	
Cu_5_Fe_1.06_S_4_	98.4	1.08483(3)	2.19419(5)	1.10617(3)	90.9
Cu_5_Fe_1.04_S_4_	99.8	1.08406(2)	2.19158(4)	1.10680(2)	87.8
Cu_5_Fe_1.02_S_4_	99.8	1.08453(2)	2.18949(3)	1.10513(2)	73.2
Cu_5_FeS_4_	99.8	1.08742(4)	2.18064(6)	1.09911(3)	64.1
Cu_5_Fe_0.98_S_4_	99.2	1.08489(3)	2.19039(5)	1.10483(3)	70.6
Cu_5_Fe_0.96_S_4_	100.0	1.08557(3)	2.19092(4)	1.10474(3)	76.3
Cu_5_Fe_0.94_S_4_	98.4	1.08601(2)	2.18992(3)	1.10379(2)	72.6

## Data Availability

The original contributions presented in this study are included in the article; further inquiries can be directed to the corresponding author.
